# SUPERA Online — Sensorial Moove: cognitive stimulation and education for active aging

**DOI:** 10.1590/1980-5764-DN-2025-0400

**Published:** 2026-04-20

**Authors:** Djessica Hilton Peixoto, Kauane Macedo Barbosa, Gabriela dos Santos, Tiago Nascimento Ordonez, Luiz Carlos de Moraes, Rosa Yuka Sato Chubaci, Beatriz Aparecida Ozello-Gutierrez, Thais Bento Lima-Silva

**Affiliations:** 1 Universidade de São Paulo, Escola de Artes, Ciências e Humanidades, Programa de Gerontologia, São Paulo SP, Brazil.; 2 Universidade Federal do Recôncavo da Bahia, Centro de Ciências da Saúde, Santo Antônio de Jesus BA, Brazil.; 3 Supera Instituto de Educação, São José dos Campos SP, Brazil.; 4 Universidade de São Paulo, Hospital das Clínicas, Grupo de Neurologia Cognitiva e do Comportamento, São Paulo SP, Brazil.

**Keywords:** Video Games, Healthy Aging, Cognition, Technology, Jogos de Vídeo, Envelhecimento Saudável, Cognição, Tecnologia

## Abstract

Education for active aging, combined with technologies and cognitive games, promotes well-being, social engagement and cognitive stimulation in older adults.

**Objective:**

The aim of this study was to analyze the effects of digital games on cognitive stimulation in older adults, combined with an educational intervention on active aging.

**Methods:**

A workshop comprising 10 weekly 90-min sessions was run. Data collection was conducted online and involved application of a sociodemographics questionnaire. The protocol included the Cognitive Function Instrument, Geriatric Anxiety Inventory, Geriatric Depression Scale-15, Satisfaction with Life Scale, Social Support Scale of Medical Outcome Study, and Lubben Social Network Scale. Sessions were conducted by two monitors who were Gerontology graduates. A total of 31 older adults took part (mean age=67; standard deviation=6.40 years). Most participants were women (96.77%) and had complete higher educational level (77.42%).

**Results:**

On the post-test, participants had a mean score for cognitive performance of 416.43 points (scale 0–1,000), most notably for impulsivity control (55.93 points), followed by reaction time (43.93) and decision-taking (41.07). Participants showed a reduction in anxious and depressive symptoms, and improved self-perceived performance in cognitive skills.

**Conclusion:**

The digital cognitive games platform proved feasible and well accepted, showing potential for expanding self-awareness about aging and promoting cognitive and psychosocial health in older adults.

## RESUMO.

A educação para o envelhecimento ativo associada ao uso de tecnologias e jogos cognitivos promove bem-estar, engajamento social e estimulação cognitiva em pessoas idosas.

**Objetivo:**

Promover o acesso a jogos de estimulação cognitiva combinados com educação sobre envelhecimento, por meio de oficinas educativas para pessoas idosas.

**Métodos:**

Foi realizada uma oficina composta de 10 sessões semanais de 90 minutos cada uma. A coleta de dados ocorreu de forma online e incluiu a aplicação de um questionário sociodemográfico. O protocolo contemplou o Instrumento de Funções Cognitivas, o Inventário de Ansiedade Geriátrica, a Escala de Depressão Geriátrica, a Escala de Apoio Social e a Escala de Redes Sociais de Lubben. As sessões foram conduzidas por duas monitoras graduadas em Gerontologia. Participaram da oficina 31 pessoas idosas (idade média=67 anos; desvio padrão=6,40 anos), sendo a maioria mulheres (96,77%) e com ensino superior completo (77,42%).

**Resultados:**

No pós-teste, a média de pontuação no desempenho cognitivo foi de 416,43 pontos (escala 0–1.000), com destaque para o controle de impulsividade (55,93 pontos), seguido por tempo de reação (43,93) e tomada de decisão (41,07). Observou-se redução nos sintomas ansiosos e depressivos, bem como melhora no desempenho autorreferido de habilidades cognitivas.

**Conclusão:**

A plataforma digital de jogos cognitivos se mostrou viável e bem-aceita, com potencial para ampliar a autoconsciência sobre o envelhecimento e promover a saúde cognitiva e psicossocial de pessoas idosas.

## INTRODUCTION

 Brazil is undergoing a rapid demographic transition, with the population aged 60 years and older increasing by 55% over the last decade^1^. This demographic shift poses significant challenges for the maintenance of autonomy, functionality, and quality of life in later life^2^. In this scenario, it becomes essential to develop actions and interventions that promote health, participation, and security for older adults, principles that underpin the concept of active aging^3,4^. 

 Aligned with the Decade of Healthy Ageing 20212030 initiative, strategies that enhance intrinsic capacity, social engagement, and cognitive functioning are considered priorities for achieving healthy and active ageing. The World Health Organization (WHO) framework emphasizes that promoting older adults’ ability to learn, make decisions, and remain socially connected contributes to their overall well-being and participation in society^5,6^. In this context, educational and preventive interventions focused on cognitive health play a pivotal role^7-9^. 

 During the ageing process, it is common to observe gradual cognitive decline, particularly in functions such as memory, attention, and executive abilities^10,11^. Cognitive stimulation interventions aim to prevent or slow down these changes by providing structured mental activities that strengthen existing neural connections and foster neuroplasticity, the brain’s ability to reorganize itself in response to new experiences and learning^12^. Evidence from randomized clinical trials and meta-analyses indicates that cognitive stimulation and training can improve global cognition, reasoning, and everyday functioning in older adults^13-15^. These benefits are particularly relevant for maintaining autonomy and preventing cognitive and functional decline^2,16^. 

 In recent years, technology has gained increasing importance in health promotion strategies for older adults. Digital cognitive training programs and computerized cognitive stimulation (CCS) platforms have been shown to enhance cognitive performance, psychosocial well-being, motivation, and engagement^17-19^. Moreover, the COVID-19 pandemic underscored the crucial role of digital inclusion in mitigating social isolation and promoting mental health among older adults^20,21^. 

 In Brazil, initiatives integrating digital tools and gerontological education remain scarce. However, preliminary findings from a study using the Supera Online Digital Platform demonstrated that participation in digital games targeting memory, attention, and executive functions was associated with reduced memory complaints and anxiety, and improved self-reported quality of life^22^. Other national studies have also highlighted the potential of digital games and educational activities to support cognitive and emotional well-being in older adults^2,9^. These findings reinforce the importance of accessible and contextually adapted interventions to promote cognitive health and active ageing among older Brazilians. 

 Therefore, the present study aimed to analyze the effects of digital games on cognitive stimulation in older adults using the Supera Online Digital Platform—Sensorial Moove, combined with an educational intervention on active aging. It was hypothesized that this combined approach would enhance cognitive functioning, psychosocial well-being, and engagement with digital technology. The relevance of this research for gerontology lies in integrating education, technology, and health promotion in an accessible and reproducible intervention, aligned with the WHO framework for healthy and active ageing^5^. 

## METHODS

### Participants

 The study sample comprised healthy older adults aged 60 years or older, with no diagnosed neurodegenerative diseases. Participants were recruited through the University of the Third Age (USP60+) program, using a mixed non-probabilistic sampling approach that combined convenience sampling and snowball sampling via social networks and participant referrals. The intervention period ran from August 2024 to November 2024. Inclusion criteria required completion of at least elementary education (≥4 years), MMSE-BRAZTEL^23^ score above 15, Geriatric Depression Scale (GDS-15)^24^ score below 6, Geriatric Anxiety Inventory (GAI)^25^ score below 10, and availability of a family member or friend to complete the Functional Activities Questionnaire^26^ (FAQ≤2). Participants with uncontrolled or decompensated chronic conditions or prior participation in cognitive stimulation programs within the previous 12 months were excluded. All participants had controlled chronic diseases and met the MOANS criteria for healthy older adults^27,28^. 

### Intervention

 The intervention involved the use of the Supera Online Digital Platform—Sensorial Moove, a digital tool developed by neuroscientists from the Faculty of Medicine of Ribeirão Preto to stimulate cognitive functions (attention, memory, motor coordination, language, and decision-making) through educational workshops. Free licenses were provided by the Supera Education Institute for this study. Ten weekly sessions of 90 min were conducted online via Google Meet, each comprising an educational stage on active aging, brain health, and lifestyle, followed by a practical stage using digital games from the Sensorial Moove platform. Undergraduate monitors in gerontology guided the sessions under supervision of the principal researcher. Attendance was recorded, with a minimum requirement of 80% presence per semester. The study aimed to pilot the platform’s usability and accessibility for healthy older adults. 

### Instruments and protocol

 Data collection was conducted online using Google Meet by gerontology undergraduates at the same institution as the research venue. Interviews took an average of 1 h and were conducted both before and after the intervention program. A sociodemographic questionnaire was used to collect data on income, education, and occupation or retirement status. The protocol entailed application of the following instruments: Cognitive Function Instrument (CFI)^29^, which measures subjective aspects of cognitive abilities; the Telephone Brazilian Mini-Mental State Exam (Braztel-MMSE)^23^, which provides an objective assessment of cognitive performance, encompassing memory, orientation, attention, language, and visuospatial ability; the Geriatric Anxiety Inventory (GAI)^25^, for identifying symptoms of anxiety in older adults; the GDS-15^24^, which quantifies the presence of depressive symptoms; the Satisfaction With Life Scale (SWLS)^30^, which measures an individual’s level of perceived contentment; the Social Support Scale of Medical Outcome Study (MOS-SSS)^31^, assessing the level of perceived support in everyday situations; and the Lubben Social Network Scale (LSNS)^32^, measuring perceived social support of older people. The inclusion of the Braztel-MMSE in this protocol provides a validated objective screener of cognitive performance, especially suited for remote assessment. 

#### Assessment protocol

**Figure 1:**
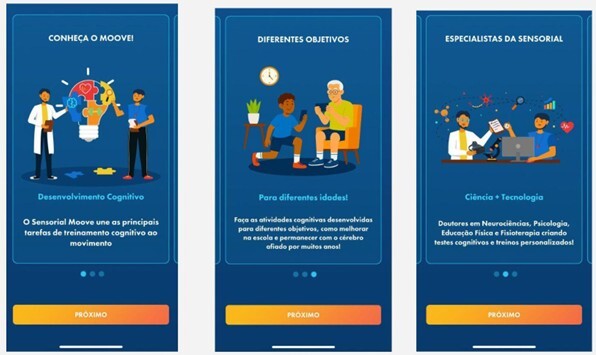
Welcome screens.

**Figure 2:**
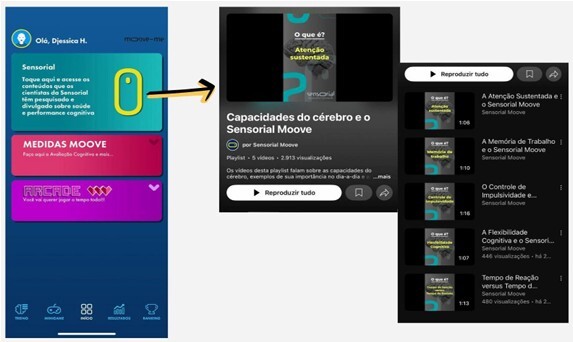
Interface for educational content and video playlists regarding cognitive domains.

**Figure 3:**
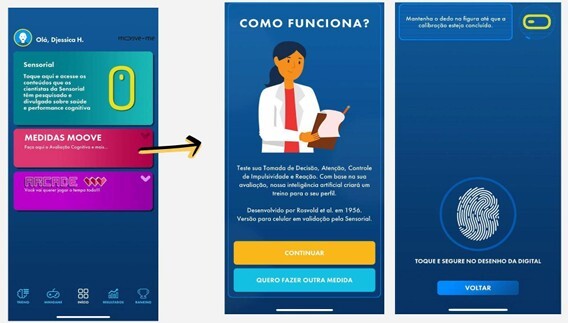
Instructions and calibration process for the cognitive performance assessment.

- CFI: is a 14-item questionnaire assessing different aspects of cognitive abilities, such as memory, language, orientation, and practical functions. Response options are "yes" (1 point), "no" (0 points), "maybe" (0.5 point) or "not applicable" (0 points), giving a total score ranging from 0 to 14. Higher scores on the instrument indicate greater perceived cognitive complaints^29^;
- GAI: is a brief self-report scale for assessing anxiety symptoms in older individuals. The GAI is used in both epidemiological studies in primary care and in clinical samples. The scale comprises five items with dichotomous responses (yes/no), focusing on non-somatic anxiety complaints^25^;
- GDS-15: is a scale designed to identify and quantify the presence of depressive symptoms in older individuals. The GDS-15 is a 15-item scale with response options of "yes" or "no." A score of 0–5 points indicates no depression symptoms; 6–10, mild-to-moderate depression; and >10 points, severe depression^24^;
- SWLS: is an instrument to measure an individual’s perceived contentment regarding different areas of their life or their life as a whole. The assessment comprises five items measuring a cognitive component of subjective well-being and involves questions probing level of enthusiasm/pleasure or discontentment/anguish an individual feels when appraising their lifestyle. Participants provide responses on a 7-point Likert scale, from 1 (strongly disagree) and 7 (strongly agree). Satisfaction with life directly impacts personal realization, influencing involvement in activities, satisfaction with vocational and career choices, besides perceived self-efficacy^30^;
- MOS-SSS: is a questionnaire to assess an individual’s perceived availability of social support for dealing with life situations. Developed originally for patients with chronic illnesses, its ease-of-use and psychometric quality has led to its wider use in other populations. The MOS-SSS contains 19 items rated on a 5-point Likert scale and assesses five types of support: emotional, informational, tangible, affectionate, and positive social interaction^31^;
- LSNS: is an instrument designed to assess perceived social support among older adults and widely used in international studies. This 18item scale measures support received from family members, friends and neighbors, and helps identify risk of social isolation. The Brazilian version of the scale is culturally and linguistically adapted for Brazilian Portuguese, retaining its conciseness and reliability^32^;
- Braztel-MMSE: Is an exam designed to assess global cognitive functioning of older adults. Domains assessed include memory, orientation, attention, language and visuospatial abilities, for a total score ranging from 0 to 30 points. Scores below 24 indicate possible cognitive impairment. The standard MMSE is traditionally applied in-person, whereas the Braztel-MMSE is a version adapted for use by telephone. The Braztel-MMSE, containing 22 items with a cut-off of 15 points, has shown good agreement with the MMSE applied in-person. This version represents a viable alternative for remote cognitive screening in different research and health settings^23^;
- Supera Online Digital Platform—Sensorial Moove—is a digital tool for stimulation and observation of cognitive functions (attention, memory, motor coordination, and logical reasoning) during educational workshops. *Supera Online Digital Platform—Sensorial Moove* is a digital resource for cognitive and motor stimulation in older adults. The system consists of an app that employs movement sensors and interactive games designed to target specific functions such as attention, memory, language, motor coordination, and decision-making, (see Figures 1, 2, and 3). During the workshops, the platform was used as a complementary tool alongside the core activities, contributing to the engagement of participants and promoting stimulation of different cognitive abilities in a fun way. The games were chosen according to the aims of each workshop session (Sensorial Life)^33^.

#### Procedures

 Participants were screened for eligibility and registered via a form capturing sociodemographic data, health status, functional autonomy, and digital literacy. Selected participants joined the Sensorial Moove workshops on a voluntary basis, aiming to enhance cognitive performance, digital literacy, and active aging engagement. Interviews and tests were conducted online using Google Meet, lasting approximately 1 h each, both before and after the intervention. Sessions had a duration of 2 h each (see Supplementary Material). 

#### Statistical analysis

 Descriptive statistics were used to characterize the sample. Non-parametric tests were applied due to non-normal data distributions. Pre- and post-intervention differences were analyzed using Wilcoxon tests, and Spearman correlations explored relationships between continuous variables. A significance level of 5% (p<0.05) was adopted. 

#### Ethics

 All participants provided informed consent. The study was approved by the Research Ethics Committee of Hospital das Clínicas, Faculty of Medicine, USP (HCFMUSP), under no. 4.357.429. Confidentiality and privacy of collected data were ensured. Declarations confirmed no conflict of interest, particularly regarding the use of a commercially available cognitive stimulation program. 

## RESULTS

 The sample comprised 31 participants with a mean age of 67 (standard deviation [SD]=6.40) years, and a predominance of females (96.77%). For marital status, most participants were married or in a civil union (48.39%), followed by separated/divorced (22.58%), and widowed (16.13%). Regarding educational level, the majority of participants had completed higher education (77.42%), a factor which may have positively influenced intervention engagement and results. In terms of family composition, 32.26% lived alone and 32.26% with a spouse, a profile which may have influenced the level of perceived social support (Table 1). 

**Table 1:** Sociodemographic data.

Variables		n	%	Mean	SD	Min.	Median	Max.
Age		31	100.00	67.00	6.40	60.00	66.00	85.00
Sex	Female	30	96.77					
	Male	1	3.23					
Marital status	Married/Civil Union	15	48.39					
	Separated/Divorced	7	22.58					
	Single	4	12.90					
	Widowed	5	16.13					
Education	Primary. complete	1	3.23					
	Secondary complete	3	9.68					
	Higher incomplete	3	9.68					
	Higher complete	24	77.42					
Living arrangements	Alone	10	32.26					
	With partner only	10	32.26					
	With children	2	6.45					
	With partner and children	5	16.13					
	With partner, children and grandchildren	2	6.45					
	With others	2	6.45					

Abbreviations: SD, standard deviation; Min., Minimum; Max., Maximum.

 The *Supera Online Digital Platform—Sensorial Moove Test*, applied at the end of the intervention, assessed different aspects of cognition and executive functions. Participants (n=14) attained a mean score of 416.43 points (SD=153.17) on overall Cognitive Performance (scale 0–1,000), most notably for impulsivity control, the specific domain with the highest mean score (55.93), followed by reaction (43.93) and decision (41.07). Mean reaction time was 350.14 milliseconds (ms) and decision time was 169.36 ms. These results demonstrate active participation in the task, with median performance indicating good potential for engagement in cognitive technologies, albeit with room for improvement in attention and response time (Table 2). 

**Table 2:** Descriptive statistics for *Supera Online Digital Platform - Sensorial Moove* test (applied post-intervention).

Variables		n	Mean	SD	Min.	Median	Max.
Supera Online Digital Platform - Sensorial Moove tests	Cognitive Performance (0–1,000)	14	416.43	153.17	203.00	434.00	645.00
	Reaction (0–100)	14	43.93	20.34	5.00	43.50	74.00
	Decision (0–100)	14	41.07	16.87	19.00	38.50	70.00
	Impulsivity Control (0–100)	14	55.93	15.97	36.00	52.00	84.00
	Attention (0–100)	14	38.21	24.25	5.00	40.00	70.00
	Reaction time (milliseconds)	14	350.14	95.97	241.00	306.50	602.00
	Decision time (milliseconds)	14	169.36	47.18	109.00	169.00	249.00

Abbreviations: SD, standard deviation; Min., Minimum; Max., Maximum.

 Results on the psychosocial and cognitive scales showed that perceived quality of life (SWLS) was largely unchanged (from 8.13 to 8.24; p=0.608), while subjective cognitive decline (CFI-14) showed a tendency toward improvement (from 4.55 to 4.00), albeit not statistically significant (p=0.083). Anxiety (GAI) and depression (GDS) also decreased slightly, again without reaching statistical significance (p>0.05). In addition to these scales, the final assessment included the Supera Online Digital Platform—Sensorial Moove test as a complementary measure of cognitive performance in a digital environment (Table 2). 

 In contrast, results were significant on the MOSSSS: significant reductions in both Tangible Support (p=0.027) and Affectionate Support (p=0.001) were observed, suggesting lower perceived support for these domains post-intervention. Total score on the MOS also decreased (from 55.61 to 46.41; p=0.041), supporting this interpretation. 

 On the LSNS, there was a significant reduction on the subdomains family (p=0.002) and neighbors (p=0.008), whereas the subdomain friendships was unchanged. Total score on the Lubben decreased from 41.10 to 35.06, a difference that was not statistically significant (p=0.332). The descriptive statistical data from the scales applied at pre and post-test are summarized in Table 3. 

**Table 3:** Descriptive statistics of scales applied at pre and post-test.

Variables		n	Mean	SD	Min.	Median	Max.	*p-value*
Satisfaction With Life Scale	SWLS Total_T0	31	8.13	1.57	5.00	8.00	10.00	
	SWLS Total_T1	17	8.24	1.60	4.00	8.00	10.00	
Cognitive Function Instrument	CFI14 Total_T0	31	4.55	3.28	1.00	3.00	14.00	
	CFI14 Total_T1	17	4.00	3.32	0.00	3.00	13.00	0.083
Geriatric Anxiety Inventory	GAI Total_T0	31	6.13	4.98	1.00	5.00	18.00	
GAI Total_T1	17	5.18	5.93	0.00	1.00	15.00	0.069
Geriatric Depression Scale	GDS Total_T0	31	2.74	3.32	0.00	2.00	13.00	
GDS Total_T1	17	2.53	3.69	0.00	2.00	13.00	0.579
Social Support Scale	Tangible Support MOS_T0	31	12.84	3.37	6.00	14.00	16.00	0.027
	Tangible Support MOS_T1	17	9.88	4.30	4.00	10.00	16.00	
	Affectionate Support MOS_T0	31	10.90	2.10	5.00	12.00	12.00	0.001
	Affectionate Support MOS_T1	17	8.47	3.02	3.00	9.00	12.00	
	Emotional Support MOS_T0	31	21.19	9.40	0.00	24.00	32.00	0.111
	Emotional Support MOS_T1	17	17.94	8.08	3.00	16.00	32.00	
	Social Interaction MOS_T0	31	10.68	4.82	0.00	12.00	16.00	0.773
	Social Interaction MOS_T1	17	10.12	3.31	6.00	8.00	16.00	
	MOS Total (0–76)_T0	31	55.61	16.78	17.00	61.00	76.00	0.041
	MOS Total (0–76)_T1	17	46.41	15.57	23.00	45.00	76.00	
Lubben Social Network Scale	Family Lubben_T0	31	18.48	5.48	7.00	18.00	30.00	
	Family Lubben_T1	17	14.00	5.27	4.00	14.00	24.00	0.002
	Neighbors Lubben_T0	31	12.06	6.08	0.00	12.00	26.00	
	Neighbors Lubben_T1	17	9.00	4.81	2.00	9.00	20.00	0.008
	Friendships Lubben_T0	31	10.55	9.49	0.00	12.00	30.00	
	Friendships Lubben_T1	17	12.06	6.07	1.00	13.00	23.00	1.000
	Lubben Total (0–90)_T0	31	41.10	14.46	25.00	36.00	85.00	
	Lubben Total (0–90)_T1	17	35.06	14.08	10.00	37.00	67.00	0.332

Abbreviations: CFI: Cognitive Function Instrument. GSD: Geriatric Depression Scale. GAI: Geriatric Anxiety Inventory. SWLS: Satisfaction With Life Scale. MOS: Social Support Scale. Lubben: Lubben Social Network Scale (LSNS-18). T0: Pre-test. T1: Post-test. SD, standard deviation; Min., Minimum; Max., Maximum.

Note: p-value for Wilcoxon test.

 Spearman’s correlation matrix (Table 4) was used to investigate associations among cognitive variables (assessed by the *Supera Online Digital Platform—Sensorial Moove* test), subjective indicators (such as perceived quality of life, mood, and social support) and measures of social support (MOS and Lubben). 

**Table 4:** Spearman Correlation Matrix.

Variable	Spearman	Cognitive Performance (0–1000)	Reaction (0–100)	Decision (0–100)	Impulsivity Control (0–100)	Attention (0–100)	Reaction time(milliseconds)	Decision time(milliseconds)
SWLS_Total_T0	rho	-0.081	0.007	-0.068	-0.122	0.437	-0.040	0.152
	p	0.782	0.982	0.818	0.678	0.207	0.893	0.674
SWLS_T1	rho	-0.212	-0.219	-0.187	-0.112	-0.191	0.219	0.218
	p	0.557	0.544	0.605	0.758	0.597	0.544	0.545
CFI14_Total_T0	rho	0.075	0.178	0.146	0.242	-0.101	-0.322	0.133
	p	0.798	0.544	0.618	0.405	0.730	0.262	0.650
CFI14_Total_T1	rho	-0.006	0.139	-0.142	-0.161	-0.177	-0.136	-0.303
	p	0.986	0.701	0.696	0.658	0.625	0.707	0.396
GAI_Total_T0	rho	0.076	0.169	0.182	0.244	0.046	-0.366	0.300
	p	0.797	0.563	0.533	0.400	0.876	0.198	0.297
GAI_Total_T1	rho	0.428	0.628	0.344	0.350	0.202	-0.569	0.337
	p	0.217	0.052	0.331	0.321	0.575	0.086	0.340
GDS_Total_T0	rho	-0.263	-0.061	-0.219	-0.058	-0.336	-0.004	0.071
	p	0.363	0.835	0.453	0.844	0.240	0.988	0.808
GDS_Total_T1	rho	0.063	0.374	-0.163	-0.019	-0.038	-0.132	0.339
	p	0.863	0.286	0.653	0.959	0.917	0.716	0.338
Tangible_Support_ MOS_T0	rho	-0.114	-0.073	-0.289	-0.067	0.006	0.187	0.253
	p	0.698	0.805	0.317	0.820	0.985	0.522	0.383
Tangible_Support_ MOS_T1	rho	0.277	0.204	0.252	0.357	0.198	−0.272	−0.049
	p	0.439	0.572	0.482	0.311	0.583	0.448	0.893
Affectionate_Support_ MOS_T0	rho	0.100	-0.033	0.180	0.131	0.289	0.067	0.336
	p	0.734	0.910	0.539	0.656	0.316	0.821	0.240
Affectionate_Support_ MOS_T1	rho	0.492	0.123	0.683	0.437	0.579	-0.185	-0.098
	p	0.148	0.734	0.029	0.207	0.079	0.608	0.787
Emotional_Support_ MOS_T0	rho	-0.126	-0.211	0.004	-0.040	0.114	0.281	0.048
	p	0.669	0.470	0.988	0.893	0.698	0.330	0.869
Emotional_Support_ MOS_T1	rho	0.116	-0.116	0.354	0.152	0.144	-0.015	0.012
	p	0.750	0.749	0.316	0.674	0.691	0.967	0.973
Social_Interaction_ MOS_T0	rho	-0.431	-0.579	-0.390	-0.390	-0.295	0.460	-0.186
	p	0.124	0.030	0.168	0.168	0.305	0.098	0.524
Social_Interaction_ MOS_T1	rho	-0.063	-0.142	0.232	0.125	-0.136	-0.120	0.119
	p	0.863	0.696	0.519	0.730	0.709	0.742	0.743
MOS_Total_(0–76)_T0	rho	-0.202	-0.302	-0.183	-0.121	-0.002	0.313	0.090
	p	0.488	0.294	0.532	0.680	0.994	0.276	0.759
MOS_Total (0–76)_T1	rho	0.236	0.043	0.430	0.297	0.226	−0.164	0.127
	p	0.514	0.907	0.218	0.407	0.531	0.650	0.733
Family_Lubben_T0	rho	-0.137	-0.064	-0.205	-0.435	0.055	0.340	-0.205
	p	0.641	0.828	0.481	0.120	0.851	0.234	0.481
Family_Lubben_T1	rho	-0.337	-0.412	-0.239	-0.172	-0.417	0.225	0.104
	p	0.340	0.236	0.506	0.635	0.231	0.533	0.774
Neighbors_Lubben_T0	rho	-0.645	-0.704	-0.440	-0.323	-0.453	0.684	-0.130
	p	0.013	0.005	0.116	0.261	0.104	0.007	0.657
Neighbors_Lubben_T1	rho	-0.232	-0.374	-0.190	-0.049	-0.095	0.334	-0.159
	p	0.518	0.287	0.600	0.893	0.793	0.345	0.661
Friendships_Lubben_T0	rho	-0.054	-0.073	0.085	0.125	-0.083	-0.141	-0.103
	p	0.855	0.805	0.773	0.670	0.777	0.631	0.727
Friendships_Lubben_T1	rho	0.262	0.012	0.378	0.098	0.288	0.104	-0.396
	p	0.464	0.973	0.281	0.789	0.419	0.775	0.257
Lubben_Total (0–90)_T0	rho	-0.587	-0.588	-0.395	-0.401	-0.397	0.553	-0.333
	p	0.027	0.027	0.163	0.155	0.160	0.040	0.245
Lubben_Total (0–90)_T1	rho	-0.067	-0.340	0.055	-0.018	-0.037	0.286	-0.261
	p	0.865	0.336	0.892	0.973	0.920	0.424	0.470

Abbreviations: SWLS, Satisfaction With Life Scale; CFI, Cognitive Function Instrument; GAI, Geriatric Anxiety Inventory; GSD, Geriatric Depression Scale; MOS, Social Support Scale (subdomains, tangible, affectionate, emotional and social interaction support); Lubben, Lubben Social Network Scale (LSNS-18), with subdomains family, neighbors and friendships; T0, Pretest; T1, Post-test; Reaction/decision time (milliseconds), measured using the *Supera Online Digital Platform—Sensorial Moove*.

Notes: rho: Spearman’s correlation coefficient. p: significance value.

 The variable "Social Interaction—MOS T0" exhibited a significant negative correlation with reaction scores on the Moove (ρ=−0.579; p=0.030). 

 Also, the variable "Neighbors—Lubben T0" showed a strong negative correlation with overall cognitive performance on the Moove (ρ=-0.645; p=0.013), most notably with the reaction domain (ρ=-0.704; p=0.005), and exhibited a positive correlation with reaction time (ρ=0.684; p=0.007). 

 Another noteworthy association was found between the variable "Affectionate Support—MOS T1" and the decision domain of the Moove (ρ=0.683; p=0.029), indicating that greater perceived affectionate support at post-test was correlated with better performance for decision-making, a central domain of executive functions. 

## DISCUSSION

 The objective of this study was to analyze cognitive and psychosocial changes among older adults participating in a cognitive stimulation program that combined the Supera Online Digital Platform—Sensorial Moove with an educational workshop on aging. 

 Although no pre–post cognitive assessment was conducted using the same instrument, the descriptive results of the Sensorial Moove Test indicated that participants engaged actively in the digital cognitive tasks, achieving intermediate-to-high performance levels in domains such as impulsivity control, reaction, and decision-making. However, due to the absence of normative data for this test and the lack of a control group, these scores should be interpreted with caution, as it is not possible to determine whether they reflect improvement, maintenance, or baseline cognitive ability. 

 Regarding psychosocial and self-report measures, no significant changes were observed in subjective cognitive decline (CFI), mood (GDS, GAI), or life satisfaction (SWLS), although small, non-significant trends toward improvement were noted. In contrast, significant reductions were found in perceived social support domains, including tangible and affectionate support on the MOS-SSS, and in family and neighbors subscales of the LSNS. These results may indicate a more critical appraisal of social networks following participation in the workshops, rather than an actual reduction in support. The intervention may have prompted participants to reflect on the quality and reciprocity of their social ties, a phenomenon previously described in studies emphasizing that perceived support depends more on relational quality than on network size^34^. 

 The correlation analyses also provided relevant insights. Lower perceived social interaction at baseline (MOS T0) was associated with faster reaction times in the digital cognitive test, while a larger neighbors network (Lubben T0) was associated with lower overall cognitive performance and slower reaction times. Conversely, greater affectionate support at post-test (MOS T1) correlated positively with decision-making performance, suggesting that positive affective bonds may contribute to better executive functioning. These findings illustrate the complexity of the relationship between social connectedness and cognitive performance, as highlighted in previous research^35^. 

 Recent evidence suggests that digital cognitive interventions can produce measurable benefits in attention, executive functions, and memory performance among older adults^36,37^. These results reinforce the potential of integrating digital tools such as Sensorial Moove into cognitive stimulation programs, particularly when aligned with structured educational approaches that encourage motivation and adherence. 

 In this context, digital inclusion and the use of cognitive training games provide cognitive stimulation, boost self-esteem, and promote psychological wellbeing of older individuals, allowing them to realize they are able to learn about and interact with the digital world. These results are supported by a recent scoping review^38^ of 33 studies, showing significant gains in memory, functioning and wellbeing in older adults that used digital and traditional cognitive training games, or exergames, as part of cognitive stimulation. Hence, this approach is congruent with the aim of education about aging, which acts as a promoter of health in fostering continued learning, active participation, and empowerment of older adults. 

 A study investigating the feasibility and effectiveness of CCT in 119 Japanese workers aged 18–65, with computerized cognitive assessments over 12 weeks, found that only 22.7% achieved the recommended training time, revealing poor intervention adherence^39^. However, in the same study, individuals who trained longer on computerized cognitive exercises showed a greater improvement in attention and executive functions. The study, akin to the present investigation, had the limitation of lacking a control group for comparative analyses. 

 A meta-analysis published in Springer Nature, encompassing 43 studies on the efficacy of cognitive training games, including *BrainHQ* in two different groups (healthy older adults and older adults with mild cognitive impairment), reported small but significant effects in the analysis of the impact of this type of intervention on the two groups. However, the analyses of near and far-transfer in the same meta-analysis showed effects only in healthy older adults, suggesting the area lacks scientific evidence to support the use of this type of training^17^. 

 Conflicting with most of the related literature, the study by Devanand et al.^40^ also tested the CCS method. A total of 105 participants with mild cognitive impairment were included, stratified by age and severity, from different centers. Participants were randomized into two groups: one that performed computerized crosswords (n=56); and another that underwent CCS (n=51) using the Lumosity app^4^. After a 78-week intervention, the group performing crosswords was found to have better cognitive development than the group completing the Lumosity program. 

 In the study of Ordonez et al.^41^ conducted a cognitive stimulation program on Japanese video game equipment adapted for use by Brazilian older adults. The results revealed improvements in cognitive performance, specifically on the memory and language domains, and there was a reduction in the level of anxiety and in the rate of memory complaints. 

 Studies on neuroplasticity show this to be fertile ground for basic research, in that it yields vital information on human aging^36,37^. In summary, this evidence shows that cognitive interventions to improve performance on mnemonic tasks are important for functioning of older individuals, such as remembering to take medications, paying bills, and preparing balanced meals, thereby contributing to independence and reducing the risk of institutionalization and mortality^34^. 

 Moreover, studies have indicated that engagement with digital technologies may influence subjective cognitive perceptions and promote greater confidence in cognitive abilities^21-24^. This finding aligns with the present results, suggesting that participation in digital environments may foster self-efficacy and cognitive engagement even when objective performance improvements are modest. 

 While the observed associations contribute to understanding the complex interplay between social and cognitive variables, the findings must be interpreted in light of the methodological limitations of this pilot study. The absence of a control group and of pre- and post-assessments with the same cognitive measures precludes causal inference regarding the effects of the intervention. Furthermore, the small sample size and short intervention period limit the generalizability of results. Future studies should include control groups of cognitively healthy older adults, standardized pre- and post-cognitive assessments, and, when possible, comparisons with normative data to better contextualize digital cognitive performance. 

 This study contributes to Gerontology by showing that workshops combining digital cognitive training games with education on aging can enhance cognitive engagement and promote psychosocial wellbeing in older adults. Participants demonstrated active involvement in cognitive tasks, and performance appeared to be influenced by social factors, such as perceived support and the characteristics of their social networks. In particular, affectionate support was positively associated with decision-making abilities, while lower baseline social interaction was linked to greater individual focus during tasks. These observations suggest that the quality of social support and the context in which cognitive stimulation occurs may play an important role in the effectiveness of digital interventions. 

 The main contribution of this study lies in demonstrating that digital tools, when applied in an accessible and contextualized manner, can foster self-esteem, engagement, and feelings of belonging, all of which are relevant for healthy active aging. Additionally, these findings can inform public policies aimed at digital inclusion of older adults and support the development of similar workshops in community settings. 

 Nevertheless, the study has limitations, including a small sample, absence of a control group, and short intervention duration, which constrain the generalization of findings and the detection of changes in some psychosocial outcomes. Future research should focus on longer interventions, larger and more diverse samples, and controlled designs, as well as explore the training of professionals to deliver interventions that combine cognitive stimulation, education on aging, and digital literacy. 

## Data Availability

The datasets generated and/or analyzed during the current study are available from the corresponding author upon reasonable request.
