# Supplementary material for: SUPERA Online — Sensorial Moove: cognitive stimulation and education for active aging
Source: Dement Neuropsychol. 2026 Apr 20;20:e20250400. doi: 10.1590/1980-5764-DN-2025-0400 (PMC13102451; doi:10.1590/1980-5764-DN-2025-0400)
Supplement: Supplementary Material [file 1980-5764-dn-20-e20250400-md01.docx]

**Supplementary Material**

Chart 1. Distribution of topics addressed in classes

| **Class** | **Topic** | **Health and Aging** | **Disease Prevention** | **Cognitive Exercises** |
| --- | --- | --- | --- | --- |
| 1 | Presentation of workshop |  |  | ✓ |
| 2 | Digital games and technologies |  | ✓ | ✓ |
| 3 | Emotions in aging | ✓ | ✓ | ✓ |
| 4 | Support networks and digital games |  |  | ✓ |
| 5 | Sleep and aging | ✓ | ✓ |  |
| 6 | Difference between cognitive stimulation games and games of chance: impacts on brain and behavior | ✓ |  | ✓ |
| 7 | Film debate – Aging, loneliness and happiness | ✓ | ✓ | ✓ |
| 8 | Emotional management with relaxation games |  | ✓ | ✓ |
| 9 | Intergenerationality and games |  |  | ✓ |
| 10 | Purpose in life and normal and non-normal events | ✓ |  | ✓ |
| 11 | Safe internet browsing |  |  | ✓ |
| 12 | Retrospective – All topics revisited |  |  | ✓ |
| **Topics addressed by Category** | | **Number of classes** | | |
| Health and Aging | | 5 | | |
| Disease Prevention | | 5 | | |
| Cognitive Exercises | | 12 | | |

Source: author elaboration (2025).
